# Identification of a Proteomic Signature for Predicting Immunotherapy Response in Patients With Metastatic Non-Small Cell Lung Cancer

**DOI:** 10.1016/j.mcpro.2024.100834

**Published:** 2024-08-29

**Authors:** Patricia Mondelo-Macía, Jorge García-González, Luis León-Mateos, Alicia Abalo, Susana Bravo, María del Pilar Chantada Vazquez, Laura Muinelo-Romay, Rafael López-López, Roberto Díaz-Peña, Ana B. Dávila-Ibáñez

**Affiliations:** 1 Liquid Biopsy Analysis Unit, Translational Medical Oncology (Oncomet), Health Research Institute of Santiago (IDIS), Santiago de Compostela, Spain; 2 Universidade de Santiago de Compostela (USC), Santiago de Compostela, Spain; 3 Galician Precision Oncology Research Group (ONCOGAL), Medicine and Dentistry School, Universidade de Santiago de Compostela (USC), Santiago de Compostela, Spain; 4 Department of Medical Oncology, Complexo Hospitalario Universitario de Santiago de Compostela (SERGAS), Santiago de Compostela, Spain; 5 Translational Medical Oncology (Oncomet), Health Research Institute of Santiago (IDIS), Santiago de Compostela, Spain; 6 CIBERONC, Centro de Investigación Biomédica en Red Cáncer, Madrid, Spain; 7 Proteomic Unit, Instituto de Investigaciones Sanitarias-IDIS, Complejo Hospitalario Universitario de Santiago de Compostela (CHUS), Santiago de Compostela, Spain; 8 Faculty of Health Sciences, Universidad Autónoma de Chile, Talca, Chile; 9 Roche-Chus Joint Unit, Translational Medical Oncology Group, Oncomet, Health Research Institute of Santiago de Compostela (IDIS), Santiago de Compostela, Spain

**Keywords:** NSCLC, immunotherapy, predictive biomarkers, circulating proteins, SWATH-MS

## Abstract

Immunotherapy has improved survival rates in patients with cancer, but identifying those who will respond to treatment remains a challenge. Advances in proteomic technologies have enabled the identification and quantification of nearly all expressed proteins in a single experiment. Integrating mass spectrometry with high-throughput technologies has facilitated comprehensive analysis of the plasma proteome in cancer, facilitating early diagnosis and personalized treatment. In this context, our study aimed to investigate the predictive and prognostic value of plasma proteome analysis using the SWATH-MS (Sequential Window Acquisition of All Theoretical Mass Spectra) strategy in newly diagnosed patients with non-small cell lung cancer (NSCLC) receiving pembrolizumab therapy. We enrolled 64 newly diagnosed patients with advanced NSCLC treated with pembrolizumab. Blood samples were collected from all patients before and during therapy. A total of 171 blood samples were analyzed using the SWATH-MS strategy. Plasma protein expression in metastatic NSCLC patients prior to receiving pembrolizumab was analyzed. A first cohort (discovery cohort) was employed to identify a proteomic signature predicting immunotherapy response. Thus, 324 differentially expressed proteins between responder and non-responder patients were identified. In addition, we developed a predictive model and found a combination of seven proteins, including ATG9A, DCDC2, HPS5, FIL1L, LZTL1, PGTA, and SPTN2, with stronger predictive value than PD-L1 expression alone. Additionally, survival analyses showed an association between the levels of ATG9A, DCDC2, SPTN2 and HPS5 with progression-free survival (PFS) and/or overall survival (OS). Our findings highlight the potential of proteomic technologies to detect predictive biomarkers in blood samples from NSCLC patients, emphasizing the correlation between immunotherapy response and the idenfied protein set.

## Highlights

• PD-L1 tumor expression yields only a 20% to 30% immunotherapy response rate, mainly due to tumor heterogeneity.
• Circulating proteins have potential as new biomarkers, reflecting tumor heterogeneity.
• The SWATH-MS strategy enables the quantification of thousands of proteins in a single run.
• The SWATH-MS strategy allowed us to identify differentially expressed blood proteins related to therapy response.
• Differentially expressed proteins between responders and non-responders will aid in treatment selection.

## Graphical Abstract

**Figure:**
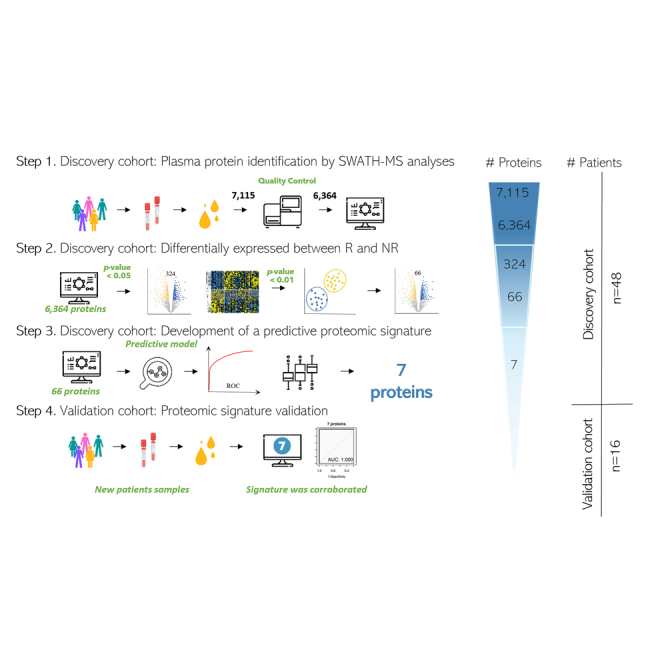

## In Brief

PD-L1 is the only biomarker that selects select patients who will respond to immunotherapy. However, it is not an unequivocal biomarker mainly due to tumor heterogeneity. In this study, we have analyzed circulating proteins from patients with Non-small cell lung cancer (NSCLC). Once analyzed, proteins were associated with response or non-response to immunotherapy, and the proteins with statistically significant differences between both groups were selected; to create a proteomic signature of response to immunotherapy treatment. The results of this work provide a proteomic signature that predicts the response to immunotherapy more accurately than PD-L1 expression, in patients with NSCLC.

## Background

Immunotherapy is one of the most promising cancer treatments due to its differential mechanism of action, long-lasting effects, and ability to improve overall survival in a percentage of patients (1). The immune checkpoint inhibitors, the most thoroughly investigated class of immunotherapy, target programmed death 1 (PD-1) receptor, its ligand (PD-L1) or cytotoxic T-lymphocyte-associated protein 4 (CTLA-4), being its objective stimulate the immune response of patients against cancer cells (2). In recent years and in comparison with chemotherapy, immune checkpoint inhibitors (ICIs) have achieved an improvement in survival rates in patients with non-small cell lung cancer (NSCLC) in both settings: monotherapy (3, 4, 5) and in combination with platinum-based chemotherapy (6, 7, 8, 9, 10). The selection of the therapeutic strategy is based on the PD-L1 expression on tumor tissue (11). However, although ICI therapies have improved outcomes for patients with NSCLC, the clinical use of immunotherapy presents new challenges related to both efficacy and safety. Regarding efficacy, a large group of patients do not respond to ICI treatment (primary resistance), whereas even in large phase III studies of ICIs combined with chemotherapy, overall response rates are 47% to 63% at best (6, 7, 8). Regarding safety, adverse reactions to ICI treatment (12, 13), as well as an accelerated progression, (denominated hyperprogression), have been reported (14).

In this context, the management of NSCLC with ICIs requires the identification of new and robust biomarkers to select patients who will benefit from immunotherapy. Liquid biopsy could allow the discovery of new biomarkers that could help clinicians to select patients who will benefit from ICI treatment (15). Numerous studies focus on potential biomarkers in the blood such as circulating tumor cells, circulating tumor DNA, circulating free DNA, exosomes, miRNA, or proteins among others due to the several advantages that present liquid biopsy compared with tumor tissue. Next-generation sequencing allows the analysis of genomes and opens the door to finding new alterations associated with response to therapies and discovering new possible therapy targets. However, typically the causes of cancer disease are multifactorial, and different approaches, including the analysis of proteomes, are required for a more comprehensive understanding. Plasma circulating proteins are an alternative biomarker that can provide information about the physical condition and status of patients. In oncology, several single serum or plasma proteins are used for the diagnosis and monitoring of cancer, such as cancer antigen 125 in ovarian cancer and the prostate-specific antigen in prostate cancer among others (16). However, nowadays the use of these single proteins should always be interpreted in conjunction with other diagnostic tests and clinical findings. Large analyses of circulating proteins could provide a wealth of information about oncogenic processes, and explore their usefulness as tumor biomarkers in response to different treatments, including immunotherapy in lung cancer (17).

In the last decades, proteomic technologies have advanced in terms of instrumentation improvements, sample preparation, and computational analyses, allowing us to identify and quantify nearly all expressed proteins in a single experiment (18). An emerging strategy named sequential window acquisition of all theoretical fragment-ion spectra–mass spectrometry (SWATH–MS) is a specific variant of the data-independent acquisition (DIA) method that combines deep proteome coverage capabilities and allows to identify and quantify circulating proteins with consistency and accuracy (19).

In the present study, we hypothesized that plasma proteins can serve as a prognostic and predictive biomarker in NSCLC patients under first-line immunotherapy. We performed a differential proteomic quantitative analysis based on SWATH–MS technology to analyze the proteome in blood samples collected from patients with advanced NSCLC prior to the start of therapy. Finally, we reported a proteomic signature of seven proteins that could predict the response to immunotherapy in advanced NSCLC patients before starting therapy, as well as at 6 and 12 weeks after starting immunotherapy.

## Methods

### Patients and Blood Sample Collection

Newly diagnosed patients with advanced NSCLC who underwent pembrolizumab therapy between January 2018 and February 2023 at the Department of Medical Oncology of Complexo Hospitalario Universitario de Santiago de Compostela were recruited. All the patients included were previously analyzed for common NSCLC alterations (*EGFR, ALK, ROS, BRAF*) to find if they present oncogenic driver mutations that can be treated with targeted therapy. A total of 64 patients with NSCLC were included in the current study. No oncogenic driver alterations were detected in their tumor tissue samples. Blood samples of 64 patients were collected before the therapy onset. In addition, 107 longitudinal blood samples were collected at 6 weeks (n = 46) and 12 weeks (n = 36) after starting the therapy and at the time of progression disease (n = 25). The best response to pembrolizumab treatment was based on RECIST 1.1, according to the following criteria: Complete response (CR), partial response (PR), stable disease (SD), or progressive disease (PD). Responders were defined as the proportion of patients with CR, PR, or SD. The study was performed in accordance with the Declaration of Helsinki (as revised in 2013). All individuals signed informed consent forms approved by Santiago de Compostela and Lugo Ethics Committee (Ref: 2017/538) prior to enrolling in the study and they could withdraw their consent at any time.

### Experimental Design and Statistical Rationale

**Fig. 1:**
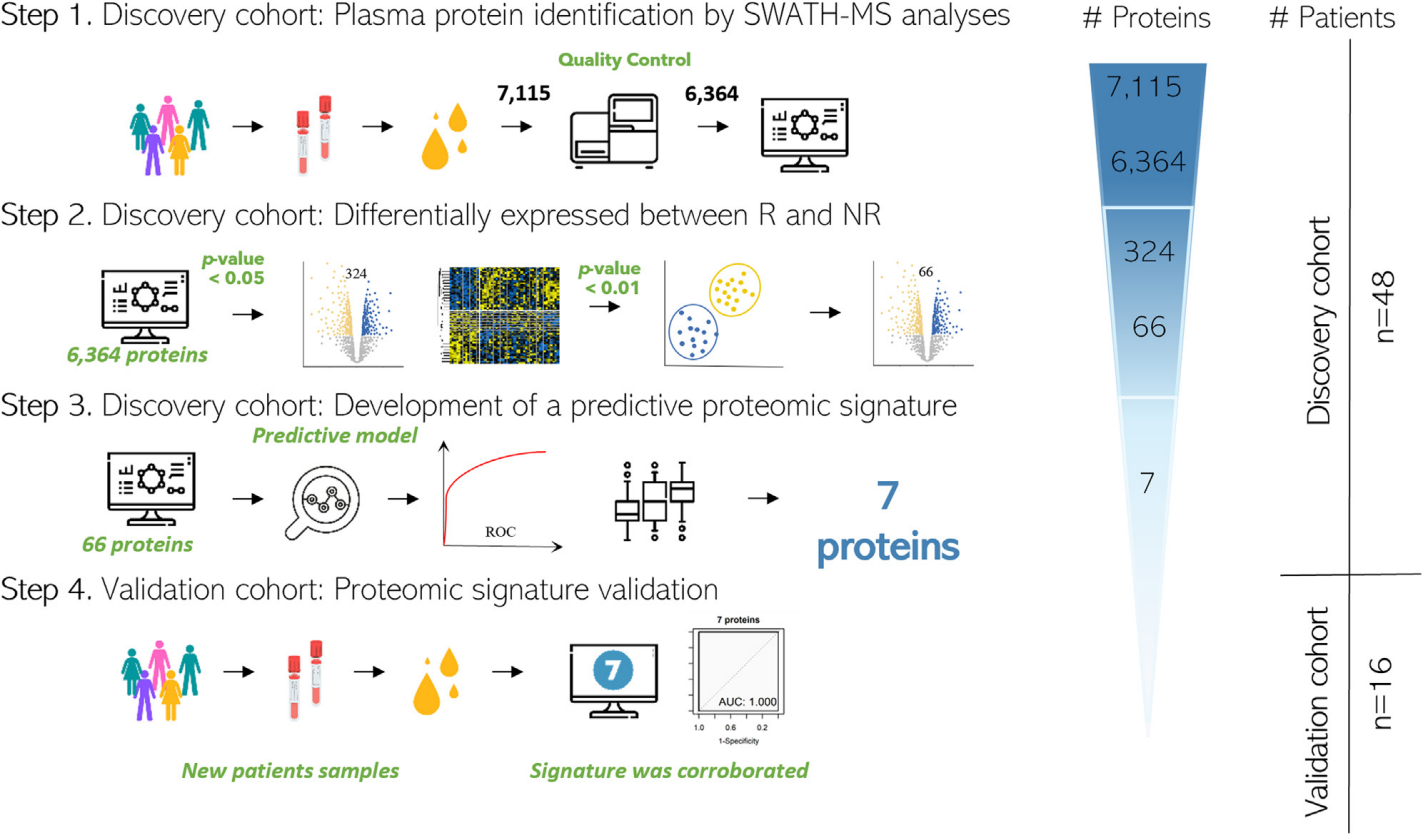
**Study design.** The proteomic workflow involved plasma extraction from blood samples of 64 NSCLC patients undergoing pembrolizumab.

The present study included different phases (Fig. 1): First, First, a Discovery cohort including 48 patients with newly diagnosed advanced NSCLC. Forty-eight plasma samples were analyzed with the SWATH-MS methodology. Initially, to ensure good reproducibility and protein recovery, samples were analyzed by triplicate. However, once this was assessed the samples were made as an individual one. Quality control to reduce missing proteins was applied, and consequently, 6334 proteins were identified. Second, patients were classified into two groups, responder and non-responder to immunotherapy as previously described. Differential analyses were performed with the purpose of identifying proteins differentially expressed between both groups. 30 hundred and 24 proteins were identified with a *p*-value <0.05. A more restrictive analysis (*p*-value <0.01) showed 66 proteins differentially expressed between both groups. Third, a protein signature was developed using bioinformatic analysis and a predictive protein signature of seven proteins was identified (detailed in Supplementary Methods). Finally, a small validation cohort including 16 newly diagnosed patients with advanced NSCLC, was employed in order to internally validate the results found in the first phase.. Samples of the validation cohort were collected in different tubes (Ethylene Diamine Tetra Acetic acid (EDTA) tubes).

### Blood Sample Processing

Ten mL of peripheral blood were obtained by direct venipuncture in CellSave tubes (Menarini, Silicon Biosystems) and processed within 96 h after blood collection for the discovery cohort. In the case of the validation cohort, peripheral blood was obtained in EDTA tubes and processed within 4 h. In both cases, plasma and cellular components were separated by centrifugation at 1600*g* for 10 min at room temperature. A second centrifugation was performed at 5500*g* for 10 min at room temperature to remove any remaining cellular debris. Plasma samples were aliquoted for storage at −80 °C until use.

### Plasma Preparation for Mass Spectrometry (MS) Analysis

#### Plasma Abundant Proteins Depletion

Aliquots of each sample (30 μl) were depleted with dithiothreitol (DTT). Fresh DTT (500 mM) was mixed with the 30 μl of human plasma samples and vortex briefly (20, 21). Then, samples were incubated until observed a viscous white precipitate that persisted for 60 min, and they were centrifugated at 18,840*g* for 20 min. Supernatants were transferred to a clean tube.

#### Isolation and Protein digestion

To make global protein identification, an equal amount of protein from all samples was loaded on a 10% SDS-PAGE gel to concentrate the proteins in a band. The band was processed as described in Supplementary Methods (22, 23).

### Protein Quantification by SWATH-MS (Sequential Window Acquisition of all Theoretical Mass Spectra)

#### Creation of the Spectral Library—Data-dependent Acquisition

To construct the MS/MS spectral libraries, the peptide solutions from the discovery cohort were analyzed by a shotgun data-dependent acquisition approach by micro-liquid chromatography-MS/MS. To get a good representation of the peptides and proteins present in all samples, pooled vials of samples from each group (basal n = 48, 6 weeks n = 35, 12 weeks n = 30, and progression disease, n = 21) were prepared using equal mixtures of the original samples for the pool spectral library building as described on Supplementary Methods.

In addition, a spectral online library called, the Human Pan-Human library, which contained 12,046 proteins was also employed in order to improve and expand the coverage of the identified NSCLC cancer plasma proteome in our library (24). The sum of the two libraries constitutes our final library, denominated NSClibrary. The mass spectrometry proteomics data have been deposited to the ProteomeXchange Consortium via the PRIDE (25) partner repository with the dataset identifier PXD042091.

#### Relative Quantification by SWATH Acquisition—DIA

For relative quantification by SWATH-MS analysis, SWATH-MS acquisition was performed on a TripleTOF 6600 LC-MS/MS system (AB Sciex). Peptides from plasma samples from all individual patients were analyzed using the DIA method. An extended method information was included in Supplementary Methods. The DIA raw data were firstly converted into mzML format filtered by SWATH Acquisition MicroApp (version 2.0) and then analyzed by PeakView (version 2.2), against our spectral NSCLibrary.

### Quality Control and Statistical Analysis

Protein values derived from the MarkerView 1.3.1 software (AB Sciex, CA, USA) were employed to perform the subsequent statistical analysis. Briefly, a quality control was first performed, during which proteins with over 30% missing values in the sample set were filtered out. Next, missing values in the remaining proteins were imputed using the RandomForest R package (26, 27), and protein values were normalized by quantile normalization. A Student’s *t* test was then applied to identify differentially expressed proteins (DEPs), with significance thresholds set at *p*-values <0.05 and <0.01 (no FDR correction). Subsequently, a predictive model was developed to differentiate the responder group from the non-responder group. The Kaplan-Meier method was used to plot survival curves, applying the log-rank test. To distinguish between low and high protein levels, ROC curves were constructed to evaluate the thresholds of baseline protein levels for survival analyses (see Supplemental Table S5 to see these threshold values). To identify the functions and relevant pathways of the DEPs, gene ontology (GO) analysis was performed using Metascape, and the Kyoto Encyclopedia of Genes and Genomes (KEGG) pathways for each group were generated and visualized with Proteomaps.

See Supplementary Methods for detailed information on each analysis. All statistical analyses were performed using GraphPad Prism version 8.0, IBM SPSS Statistics version 25.0, and R version 4.1.1.

## Results

### Patient Characteristics

**Fig. 2:**
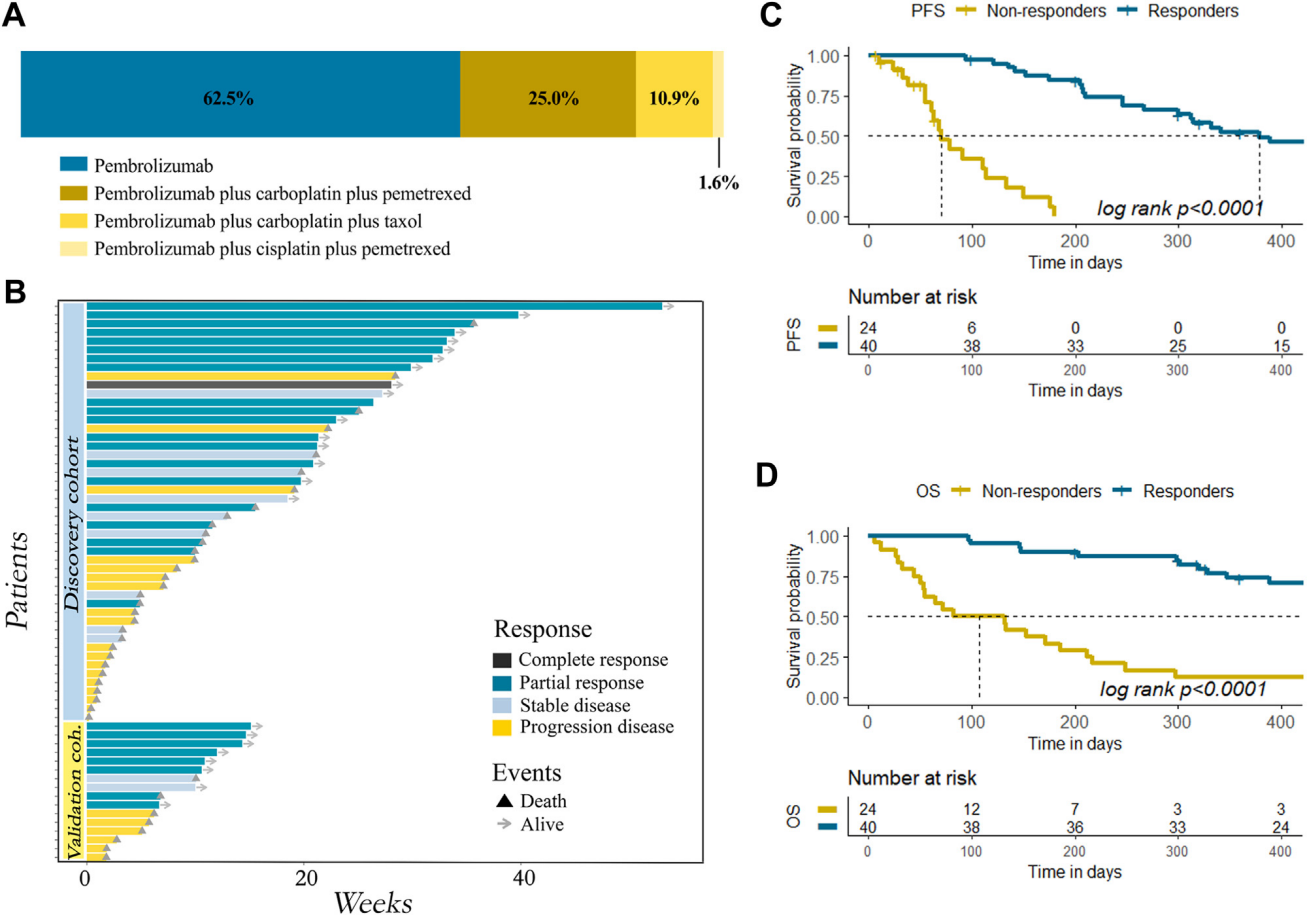
**Clinical data and treatment outcomes in our NSCLC cohort.***A*, percentage distribution of patients with different treatment regimens. *B*, Swimmers’ plot on patients showing the response of therapy. The total length of each bar indicates the duration of survival from the diagnoses. *C-D*, Kaplan-Meier plots show highly significant differences between responders and non-responders to pembrolizumab treatment in PFS (*B*) and OS (*C*).

A total of 64 patients with newly diagnosed NSCLC who received pembrolizumab treatment as monotherapy (n = 40) or in combination with chemotherapy (n = 24) as first-line treatment were included in the study (Fig. 2 and Table 1). Figure 2*A* shows the percentage of patients treated with each regimen. The most common chemotherapy regimen was carboplatin plus pemetrexed (n = 16). Patients were enrolled in two different sub-cohorts: a discovery cohort with 48 patients with NSCLC and a validation cohort with 16 patients with NSCLC. The validation cohort was included to evaluate the possible impact of the blood collection tubes. In global terms, the mean age was 64.7 years (range: 45–80), most of the patients were males (75%), current or former smokers (87.5%), and had tumors with adenocarcinoma histology (81.25%) (Table 1). The Eastern Cooperative Oncology Group performance status of 0 accounted for 17.2% of patients. Regarding the clinical characteristics of R and NR patients, it is important to mention that 61.1% of NR had more than two metastatic sites versus 16.7% for the R group (Fisher test; *p*-value< 0.01). In addition, the R group showed significantly better PFS (19.27 versus 2.3 months, respectively) and OS (35.6 versus 3.4 months, respectively) than NR (Fig. 2, *C*–*D*). In the validation cohort, the analyzed samples were taken from 16 patients with advanced NSCLC undergoing first-line pembrolizumab treatment in monotherapy (n = 8) or combination with chemotherapy (n = 8) (Table 1). 62.5% of patients were classified as R (including stable disease (n = 2) and partial response (n = 8)) and 37.5% of patients were denominated NR (progressive disease; n = 6). The validation cohort presented similar characteristics to the discovery cohort, except for sex distribution (Table 1). Similar to the previous cohort, R and NR presented significant differences in the PFS and OS rates.

**Table 1:** Patient demographics and clinical characteristics at baseline

Clinical characteristics	Discovery cohort (n = 48)			Validation cohort (n = 16)		
	R (n = 30)	NR (n = 18)	*p* value	R (n = 10)	NR (n = 6)	*p* value
	N (%)	N (%)		N (%)	N (%)	
Mean age ± SD, range	64.3 ± 8.2, 50–80	61.9 ± 9.5, 45–78	0.36	71.5 ± 5.4, 60–77	63.2 ± 7.6, 51–74	**0.02**
Sex						
Female	6 (20.0)	5 (27.8)	0.72	1 (9.1)	4 (66.7)	**0.03**
Male	24 (80.0)	13 (72.2)		9 (90.9)	2 (33.3)	
Smoking						
Smoker/Former smoker	25 (83.3)	16 (88.9)	0.70	9 (90)	6 (100)	
Never	5 (16.7)	2 (11.1)		1 (10)	0 (0.0)	1
ECOG PS						
0	8 (26.7)	1 (5.6)	**0.04**	1 (10)	0 (0.0)	
1	21 (70.0)	13 (72.2)		10 (90)	4 (66.7)	0.11
2	1 (3.3)	4 (22.2)		0 (0.0)	2 (33.3)	
Histology						
Adenocarcinoma	26 (86.7)	16 (88.9)		6 (60)	4 (66.6)	0.28
Squamous cell carcinoma	3 (10.0)	2 (11.1)	1	4 (40)	1 (16.7)	
Others	1 (3.3)	0 (0.0)		0 (0.0)	1 (16.7)	
Number of metastatic sites						
≤2	25 (83.3)	7 (38.9)	**<0.01**	7 (70)	4 (66.6)	1
>2	5 (16.7)	11 (61.1)		3 (30)	2 (33.3)	
Pembrolizumab treatment						
Monotherapy	17 (56.7)	15 (83.3)	0.07	5 (50)	3 (50.0)	1
Plus chemotherapy	13 (43.3)	3 (16.7)		5 (50)	3 (50.0)	
PFS (median days)	19.3 months	2.3 months	**<0.001**	10 months	2.7 months	**<0.001**
OS (median days)	35.6 months	3.4 months	**<0.001**	NA	3.9 months	**<0.001**

Bold text indicates statistically significant results (*p* value <0.05).

Abbreviations: NR, nonresponders; R, responders; SD, standard deviation.

### Global Proteomic Quantitative Analysis of NSCLC Blood Samples

One hundred 71 samples from 64 NSCLC patients were analyzed using high-resolution mass spectrometry to identify as many proteins as possible in the plasma samples. First, to obtain quantitative protein information from SWATH-MS, we generated an extensive spectral library, based on pooled samples and the online Human Pan-Human library. Thus, our final spectral library, called NSCLibrary, contained 7115 unique proteins. Next, we analyzed each separated sample against our spectral NSCLibrary and we extracted quantitative data from the discovery cohort, a set of 18 non-responder and 30 responder patients. An average of 6483 proteins/sample using a minimum of 10 peptides/protein and a 1% false discovery rate (FDR) on the peptide and protein level were found. Next, after sample normalization by quantile method (Supplementary Fig. S1) and sample filtering, we quantified a total of 6364 unique proteins, of which 2418 had observations in all samples. In total, 305,472 values were analyzed where 3,78% of them were imputed. In a second step, 37 plasma samples from 16 patients with NSCLC corresponding to the validation cohort were also analyzed using the same protocol, obtaining an average of 6325 proteins/sample.

### Plasma Proteome in Patients With NSCLC is Associated With Immunotherapy Response

**Fig. 3:**
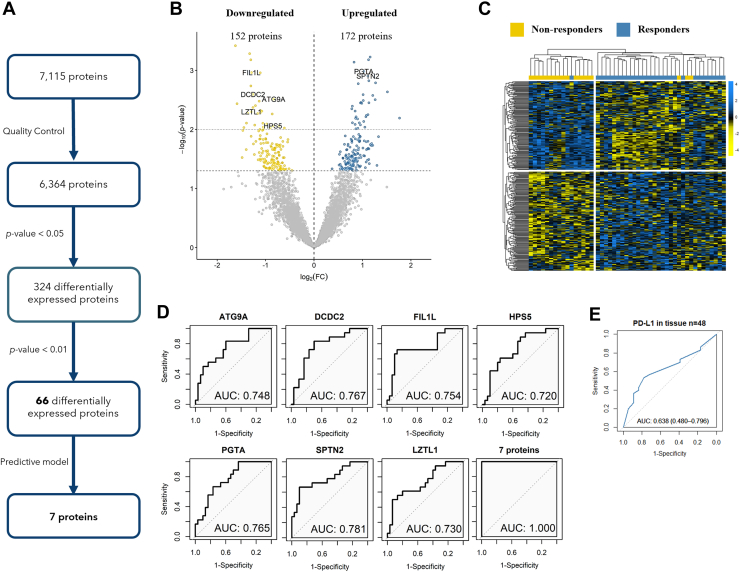
**Discovery cohort analyses.***A*, number of proteins detected by SWAT-MS and the posterior analyses. *B*, Volcano plot of differentially expressed proteins at baseline, showing the seven proteins of our predictive model. Upregulated proteins are represented in *blue* while downregulated proteins are represented in *yellow*. (C) Heatmap of 324 differentially expressed proteins at baseline (*p*-value <0.05), that discriminate between R and NR patients to pembrolizumab therapy. *D*, ROCs curves of each protein and the combination in the discovery cohort. *E*, ROC curve analyses of the PD-L1 expression on tumour tissue. Abbreviations: AUC, area under the curve; FC, foldchange.

Aiming to identify a protein signature associated with response to immunotherapy, we compared the plasma protein expression profile of 30 R and 18 NR from the first cohort of 48 patients with NSCLC. Importantly, there were no differences in the proteins’ coverage between R and NR group (Supplementary Fig. S2). We applied Student’s t tests with a nominal *p*-value cut-off of 0.05 (*p*-value <0.05) and we identified 324 DEPs (Fig. 3*A*). 5.28% protein values from these 324 DEPs were imputed. Of them, 172 proteins appeared upregulated while 152 were downregulated in R versus NR (Fig. 3B-C). We conducted functional enrichment analyses with the 324 DEPs found between R (172 proteins) and NR (152 proteins) to identify the biological functions and relevant pathways involved in the sensitivity to pembrolizumab therapy. Global analyses have not reported clear results about the GO process involved with the 324 DEPs in conjunction (Supplementary Fig. S3). Next, briefly, GO pathways comparing both groups reported that the 172 proteins more present in the R group were associated with different processes such as metabolic and cellular processes or immune system processes, in contrast with the pathways obtained in the NR group. Of note, the immune system process (GO:0002376) was enriched exclusively in the R group (Supplemental Fig. S3). In addition, proteomaps clustering the differentially expressed proteins according to their Genes and Genomes KEGG pathway annotations (Supplemental Fig. S3), reported discrete differences between both groups. Despite the results should be taken with caution, the R group seems to be characterized by higher levels of metabolic proteins than NR.

### Identification and Development of a Model that Enables the Prediction of Response to Pembrolizumab

To develop a model that allows us to predict immunotherapy response we selected the DEPs (n = 66) with a tight *p*-value (*p*-value <0.01) between R and NR patients. Thirty-six of these proteins were more expressed in the R group, while 30 were more expressed in the NR group, performing good discrimination between both groups (Supplemental Fig. S4). Then, we aimed to create a proteome signature that could accurately distinguish between these patient groups with a minimal number of proteins using stepwise forward variable selection method based on Akaike Information Criterion. Thus, a predictive model composed of seven proteins was selected to effectively discriminate between R and NR. Of these seven selected proteins, five of them were more expressed in the NR group and two of them were more expressed in the R group. The characteristics of the selected proteins are specified in Supplementary Tables S1 and S2 and Supplementary Fig. S4*C*.

Single proteins showed a good discriminatory power to distinguish responders from non-responders (AUC = 0.72–0.78). In addition, in ROC analyses their combination showed a high AUC (AUC = 1). (Fig. 3*D*), in contrast with the results obtained with the PD-L1 expression on tumour tissue (AUC = 0.638). PD-L1 expression showed a lower predictive value than our proteins and their combination (Fig. 3, *D* and *E*).

**Fig. 4:**
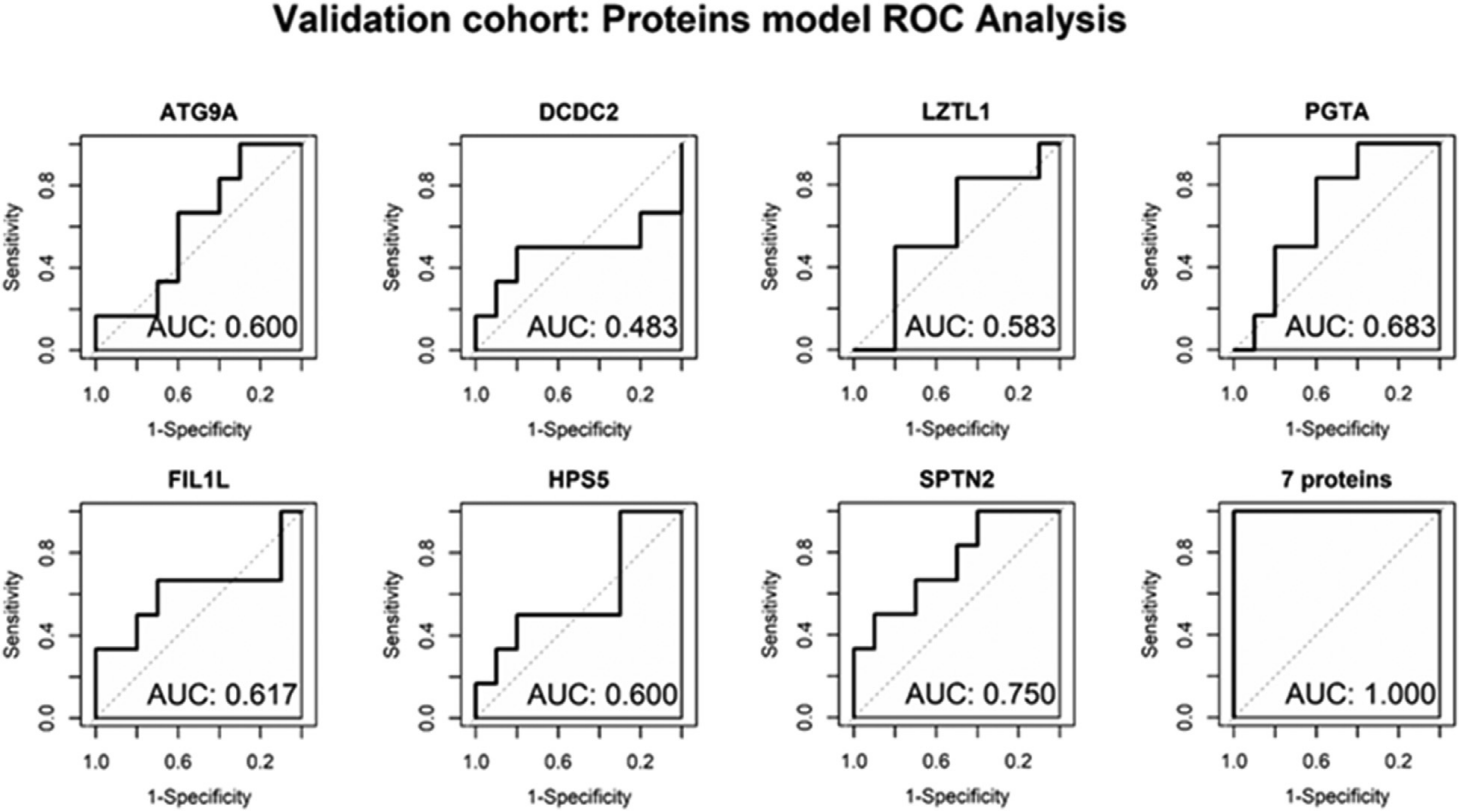
**ROC curves of each protein and the combination in the validation cohort**.

Next, an internal validation was performed using an additional cohort of 16 patients with NSCLC (Table 1). Blood samples were collected in EDTA tubes instead of CellSave tubes but analyzed with the same methodology as the discovery cohort. The protein levels of our proteomic signature were analyzed between R (n = 10) and NR (n = 6). No significant differences were found between both groups, likely due to the small size of the cohort. Nevertheless, ROC analyses were conducted to combine the seven proteins of the model, which showed a perfect discrimination rate (AUC = 1) in the validation cohort (Fig. 4). The AUC and confidence intervals for each protein model stratified by cohort type and histology are specified in Supplementary Tables S3 and S4, respectively.

### The Proteome Signature was Associated With Progression-Free Survival and Overall Survival in Patients With NSCLC

**Fig. 5:**
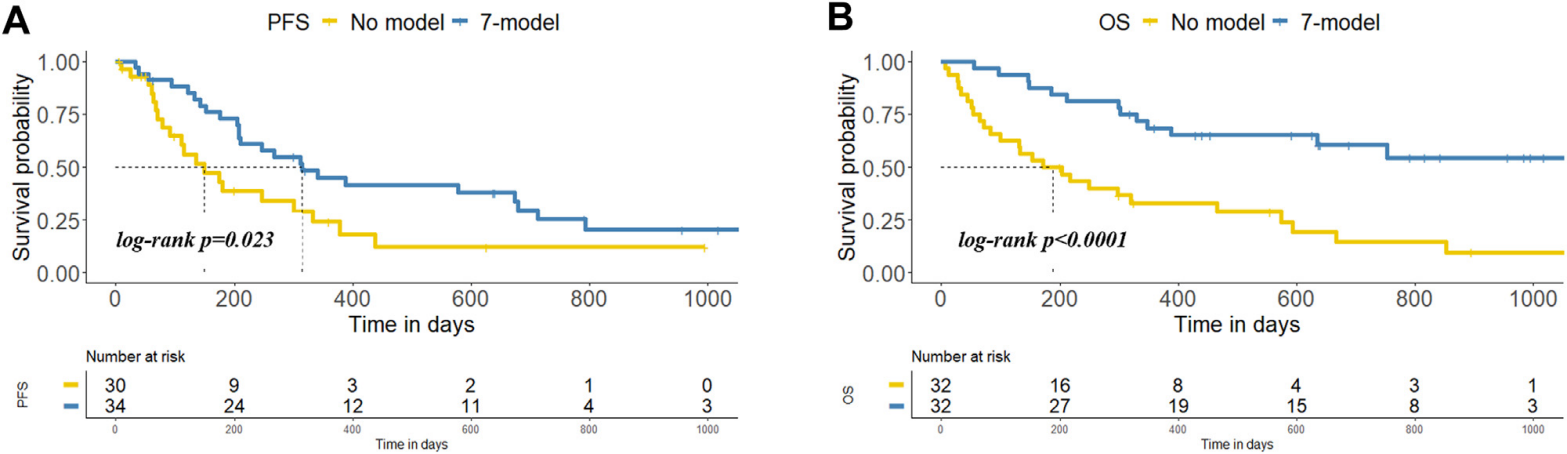
**Kaplan-Meier survival analysis using the 7-protein model.** Kaplan-Meier survival analysis of the 7-protein model for PFS (*A*) and OS (*B*) showed significant associations between protein levels before the start therapy and prognosis in metastatic NSCLC under immunotherapy regimens.

To explore the prognostic significance of the seven selected proteins, we investigated their expression levels in association with PFS and OS in our global cohort. Single protein survival analyses were performed. For this purpose, patients were dichotomized into high and low protein levels based on ROC analyses. The threshold protein levels (for PFS and OS analyses) are summarized in Supplementary Table S5. Protein levels higher than the threshold were considered "High", while those lower than the threshold were considered "Low". Kaplan-Meier curve analysis showed that four protein levels were associated with PFS and/or OS: Low levels of ATG9A and high levels of SPTN2 were significantly associated with longer PFS (log-rank *p* < 0.001 and log rank *p* = 0.017 for ATG9A and SPTN2, respectively) in NSCLC patients. Additionally, low levels of HPS5 showed a trend towards being associated with longer PFS (log-rank *p* = 0.054) and were significantly associated with longer OS (log-rank *p* < 0.001). Low expression levels of DCDC2 were also significantly associated with longer OS in our global cohort (log-rank *p* < 0.01) (Supplementary Fig. S5). No association with the patient’s survival was found for the proteins PGTA, FIL1L, and LZTL1 (Supplementary Fig. S5). Finally, we investigated the association of our 7-protein model with survival rates in the global cohort. For this analysis, patients were stratified into two groups: the 7-model group, comprising individuals with at least four proteins conforming to the model distribution based on thresholds previously mentioned (Supplementary Table S5), and the no-model group, consisting of those with fewer than four proteins conforming to the model distribution. To summarize, the patients were included in the 7-model group if four or more conditions were present at baseline: ATG9A protein levels low, DCDC2 low, FIL1L low, HPS5 low, LZTL1 low, PGTA high and/or SPTN2 high. The 7-model group demonstrated longer PFS and OS compared to the no-model group (log-rank *p* = 0.023 and log-rank *p* < 0.0001, respectively) (Fig. 5).

### Dynamics of the Proteome Signature during Pembrolizumab Therapy

**Fig. 6:**
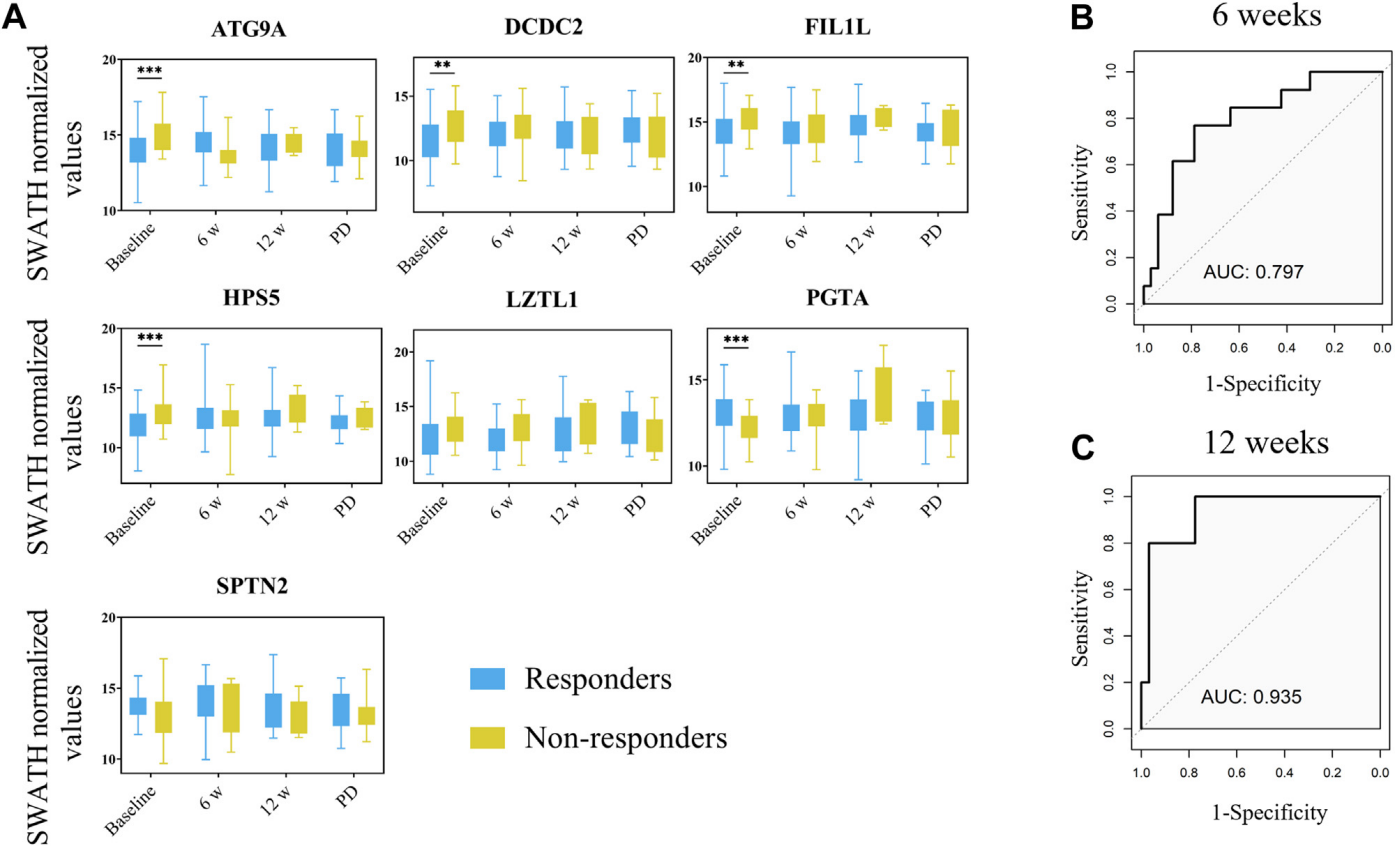
**Dynamics of the 7-protein model during treatment in NSCLC patients.***A*, Protein levels of ATG9A, DCDC2, FIL1L, HPS5, LZTL1, PGTA and SPTN2 were measured at different time points during the immunotherapy regimen. Protein levels refer to the SWATH normalized values. *B-C*, ROC analyses with the protein model at different time points during the immunotherapy regimen (at 6 weeks and at 12 weeks after start therapy). PD, progressive disease.

Next, 107 plasma samples collected at different time points were analyzed to investigate whether our protein model undergoes changes during immunotherapy treatment. A total of 46 samples were analyzed at 6 weeks, 36 at 12 weeks, and 25 at the time of disease progression. The results obtained showed that the protein levels did not show significant changes during different time points of the immunotherapy regimen, neither in the overall cohort nor when separated by response groups (Fig. 6*A*).

ROC curves analyses at 6 weeks and at 12 weeks were also performed. In all time points, our model allows us to predict therapy response with an AUC >0.70 (Fig. 6*B*). Importantly, at 12 weeks after start therapy, a good discriminatory rate was found (AUC = 0.935) (Fig. 6*C*), suggesting the potential value of the model to determine the response to immunotherapy also during the treatment.

## Discussion

Immunotherapy, in particular ICIs, has improved survival rates in patients with cancer, including those with NSCLC. However, it remains unclear how to select those patients who will respond to this therapy. PD-L1 expression on tumor tissue has been reported as a possible biomarker, but some patients without PD-L1 expression also respond to ICIs (28, 29). Tumor mutational burden (TMB), which refers to the number of somatic mutations in tumors, has recently emerged as another possible biomarker in the immunotherapy field. Nevertheless, there are several challenges for the clinical implementation of TMB, especially in standardizing detection methods and appropriate thresholds by tumor type (30, 31). Microsatellite instability, usually based on immunohistochemistry analyses for four mismatch repair proteins, may represent a novel biomarker to select patients who will benefit from ICI therapy in several cancer types (32); however, this alteration is scarcely present in lung cancer (33). The search for new predictive and prognostic biomarkers for ICI therapies will improve patient selection. Proteomics, the study of the entire set of proteins expressed in a cell, tissue, or individual, has become an important field in molecular sciences, as it provides valuable information on the identity, expression levels, and modification of proteins (34). In this work, we hypothesized that plasma proteome analyses could have predictive and prognostic value in newly diagnosed NSCLC patients who started pembrolizumab therapy. We employed SWATH-MS, a new technology that allows the detection of tens of thousands of peptides in a single injection and enables the identification and quantification of circulating proteins. Previously, several studies in patients with breast (35), bladder (36), and endometrial cancer (37), among others, have shown the potential of this emerging technology.

Importantly, our study provides a dataset that might serve the scientific community as a resource of clinical proteomic data in lung cancer samples, previously reported in melanoma (38) and ovarian cancer (39, 40).

Interestingly, with regard to different treatment regimens, our cohort results show that patients treated with pembrolizumab in combination with chemotherapy appear to achieve a more favorable response compared to those receiving immunotherapy alone (*p* = 0.07) (Table 1). This suggests that the addition of chemotherapy may have a positive impact on patient outcomes in our cohort. Nevertheless, several factors should be taken into account: a) the number of patients treated with both regimens is unbalanced, 42 patients were treated with monotherapy regimen while 16 were treated with immunotherapy plus chemotherapy; b) in our country, the use of immunotherapy is reserved for patients with PD-L1 >50%. At the time of recruitment, the combination of chemotherapy and immunotherapy was not allowed in this subgroup. In the literature, OS results in the PD-L1 >50% subgroup are similar with the use of immunotherapy as monotherapy or in combination, but the percentage of responses is higher with the combination; and c) finally, the combination of chemotherapy with immunotherapy reduces the number of patients with rapid progression compared to patients treated with immunotherapy as monotherapy."

More importantly, the analyses allowed us to identify 324 DEPs between R and NR, showing the potential value of proteomic analysis to identify differences between both groups. Previously our group has employed the methodology here described to study the red blood cell population proteome in patients with breast cancer (41). A similar study focusing on breast cancer reported that this approach was useful in determining DEPs in serum samples associated with the response to neoadjuvant chemotherapy. The authors of this study described three proteins especially correlated with resistance to this therapy (35). Some other studies focused on patients with melanoma, also reported the potential of proteomic technologies to identify DEPs and predict therapy response in tissue (38) and plasma samples (17). Regarding the enrichment analyses of biological functions and pathways, and even though the results should be interpreted with caution, we observed a high degree of variability in GO processes among the 324 DEPs between R and NR groups, indicating the significant heterogeneity present in the plasma samples. These samples can reflect both individual characteristics and tumor features, as well as the tumor microenvironment (42). Next, comparing both groups, we found that the process involved in the immune system was enriched exclusively in R, as expected given the known role of the immune system in response to ICI therapies. More analyses should be performed to confirm these results. In KEGG pathways, R appear to have higher levels of metabolic proteins than NR patients, but caution should be exercised when interpreting the results due to the lack of statistical significance. Nevertheless, our findings are consistent with those of Harel *et al*. (38) in their study of patients with melanoma. Their research aimed to investigate why most cancer patients do not respond to immunotherapy by profiling the proteome of tumor tissue samples from advanced melanoma patients undergoing either TIL-based or anti-PD1 immunotherapy. They found 414 DEPs and 636 DEPs between responder and non-responder patients in the TIL and anti-PD1 immunotherapy cohort, respectively. Pathway analyses revealed that responders had higher oxidative phosphorylation and lipid metabolism, which increases melanoma immunogenicity and sensitivity to T cell-mediated killing. These findings suggest that the metabolic state of melanoma may play a role in the response to immunotherapy and could lead to improved therapeutic responses in the future. A similar process may be occurring in our patients with NSCLC. The same author recently presented a study that showed the capability of two pro-inflammatory chemokines (CXCL8 and CXCL10) along with sex and age clinical parameters, to predict clinical outcomes with an AUC of 0.79. He and his collaborators managed to screen 760 to 1000 proteins in each sample while we were able to find an average of 6483 proteins/sample by our methodology, proving the depth of SWATH-MS for biomarker discovery. Interestingly from all proteins analyzed in both studies, two are common (43).

Next, a predictive model to identify proteins that can be associated with the response to immunotherapy treatment was developed, showing that the combination of seven proteins can predict the response to immunotherapy with high sensitivity and specificity, in a discovery cohort and, in addition, in a validation cohort. Regarding the proteins, five of them were more expressed in NR than R group: ATG9A, DCDC2, HPS5, FIL1L, and LZTL1. The ATG9A protein is a transmembrane protein that plays an important role in the formation and regulation of autophagosomes, which are cellular structures involved in the process of autophagy. This protein is localized on the membranes of the endoplasmic reticulum and Golgi (44). A recent study characterizes novel functions of ATG9A as component of a TNF-induced cell death checkpoint (45), In addition, it has been reported how NDRG1 overcomes the resistance to immunotherapy of pancreatic patients through inhibiting ATG9A-dependent degradation of MHC-1. We have observed low expressions of ATG9A in our cohort of patients related to the response of the immunotherapy that can be correlated in somehow the inhibition observed in the aforementioned (46).

DCDC2 is a protein expressed in the brain that has been linked to the development of cognitive skills such as reading and language processing. DCDC2 has been found to be localized to the cilia of neurons in the developing brain, where it is involved in the control of ciliogenesis and ciliary length (47, 48). In addition, DCDC2 seems to play a role in the inhibition of the canonical Wnt signaling pathway (49).

Notably, a recent work that developed novel prognostic biomarkers based on gene expression levels found a significant upregulation of the gene DCDC2 among others (50). HPS5 is a protein that plays a crucial role in the biogenesis and function of lysosome-related organelle and may be involved in the regulation of general functions of integrins (51). On the other side FIL1L is a protein that acts as a regulator of the antiangiogenic activity on endothelial cells. The overexpression in endothelial cells leads to the inhibition of cell proliferation and migration and an increase in apoptosis (52). In the oncology field, FIL1L has been previously reported as a protein downregulated in ovarian cancer (53). In addition, it has been shown that the knockout of FILIP1L gene leads to pulmonary adenoma formation in mice; suggesting that repression of *FILIP1L* is an important event during initiation or early progression of lung adenocarcinomas (54) LZTL1 regulates ciliary localization of the BBSome complex (55) and may have tumour suppressor function in several primary cancer types (56).

On the other hand, PGTA and SPTN2 showed higher levels in R than NR group in our study. Both proteins have been less explored. Regarding PGTA it is believed to play a role as a catalytic protein (57). In contrast, SPTN2 probably plays an important role in neuronal membrane skeleton.

To our knowledge, this is the first time that these proteins have been associated with immunotherapy response in patients with NSCLC.

Importantly, our proteomic signature (considering the combination of the seven proteins or the use of single proteins) showed a stronger value to predict immunotherapy response than the actual biomarker, the PD-L1 expression in tumour tissue. Hence, the proposed model could be useful to select which patients can be treated with immunotherapy, and in which patients, an alternative therapy should be considered. In addition, survival analyses showed that low levels of ATG9A, DCDC2, and HPS5 were associated with longer PFS or OS rates, while low levels of SPTN2 were significantly associated with worse OS.

Additionally, longitudinal samples were analyzed in the global cohort to investigate the value of the predictive model during immunotherapy. Although no significant differences were found between the R and NR groups during therapy, ROC analyses at 6 and 12 weeks showed a good discriminatory rate between the two groups. The determination at 6 weeks could assist clinicians in detecting an early response to therapy in patients with NSCLC or in cases where sample extraction was not possible at baseline. Interestingly, at 12 weeks a higher AUC was found with our model, which could help clinicians determine treatment response in cases where patients experience pseudoprogressions through imaging techniques (58), suggesting the possibility to use in a complementary manner both approaches during ICIs therapies. However, it's important to note that larger studies should be conducted to provide a more detailed and conclusive interpretation.

Finally, some limitations should be considered in the present study. First, our study included patients with diverse histological subtypes and under-treated with different immunotherapy regimens. This heterogeneity in our cohort, comprising a relatively small number of patients with histologies other than adenocarcinoma, as well as the wide range of treatment regimens, underscores the importance of future investigations focusing on more homogeneous patient populations to facilitate more definitive conclusions. Second, a validation cohort with more patients should be employed to confirm the predictive value of our model and its utility during immunotherapy. Next, to implement a technology based on quantifying the seven protein levels into clinical routine, a simpler approach such as an ELISA assay would be more appropriate. Thus, an analytical validation employing an ELISA assay should be performed.

In summary, the results of this work confirmed that circulating proteins can be used as predictive and prognostic biomarkers in patients with metastatic NSCLC and allow us to detect a proteomic signature of seven proteins (ATG9A, HPS5, DCDC2, PGTA, FIL1L, LZTL1, and SPTN2) which enables the selection of those patients who will benefit from anti-PD1 immunotherapy.

## Data Availability

Data are available via ProteomeXchange with identifier PXD042091.

## Supplemental data

This article contains Supplemental Data (20, 21, 24, 25, 41, 59, 60, 61, 62, 63, 64, 65, 66, 67, 68).

## Ethics Approval and Consent to Participate

This study was approved by the Santiago de Compostela and Lugo Ethics Committee (Ref: 2017/538). All individuals gave written informed consent to participate.

## Conflict of Interest

The authors declare the following financial interests/personal relationships which may be considered as potential competing interests:

JGG receives honoraria for consultancies from AstraZeneca, Boehringer-Ingelheim, Bristol-481 Myers Squibb, MSD, Novartis, Roche, Sanofi, and Takeda: honoraria for educational activities 482 from AstraZeneca, Bristol-Myers Squibb, MSD, Novartis, Pierre-Fabre, Roche, Sanofi and 483 Takeda; and he receives honoraria for travels and accommodations from AstraZeneca, Bristol-484 Myers Squibb, MSD, Roche, Sanofi and Takeda. LLM receives honoraria for lectures from Pfizer, 485 Boehringer, Novartis, AstraZeneca, Sanofi, Bristol, MSD, Takeda, Jansen; for advisory board 486 from Sanofi, Lilly, Novartis, Boehringer, Amgen and receives support for attending meetings 487 from MSD and AstraZeneca, outside the submitted work. RLL reports grants and personal fees 488 from Roche, Merck, AstraZeneca, Bayer, PharmaMar, and Leo and personal fees and nonfinancial 489 support from Bristol-Myers Squibb and Novartis, outside of the submitted work.

## Supplementary Data

Supplementary Tables and Figures

100 Variable Swath-MS Windows
