# Supplementary material for: Identification of a Proteomic Signature for Predicting Immunotherapy Response in Patients With Metastatic Non-Small Cell Lung Cancer
Source: Mol Cell Proteomics. 2024 Aug 29;23(10):100834. doi: 10.1016/j.mcpro.2024.100834 (PMC11474190; doi:10.1016/j.mcpro.2024.100834)
Supplement: Supplementary Tables and Figures [file mmc1.docx]

**Supplementary Materials**

## Methods

### Plasma preparation for mass spectrometry (MS) analysis

*Plasma abundant proteins depletion*

Aliquotes of each samples (30 μl) were depleted with dithiothreitol (DTT). Fresh DTT (500 mM) was mixed with 30 μl of human serum samples and vortex briefly[1] [2]. Then the samples were incubated until to observe a viscous white precipitate that persist for 60 min, then centrifuge at 18,84 g for 20 min. Supernatants were transferred to a clean tube for protein digestion.

*Isolation and proteins digestion*

An equal amount of protein from all samples were loaded on a 10% (sodium dodecyl sulfate polyacrylamide) SDS-PAGE gel .The run was stopped as soon as the front had penetrated 3 mm into the resolving gel[3] The protein band was detected by Sypro-Ruby fluorescent staining (Lonza, Switzerland), excised, and processed for in-gel manual tryptic digestion, as described elsewhere [3] Proteins were reduced with DTT 10mM in Ambic 40mM and alkylated with 55 mM iodoacetamide in 50 mM ammonium bicarbonate. Then, the gel pieces were rinsed with 50 mM ammonium bicarbonate in 50% methanol dehydrated by addition of acetonitrile and dried in a SpeedVac. Modified porcine was added to the dry gel pieces at a final concentration of 20 ng/μl in 20mM ammonium bicarbonate, incubating them at 37 °C for 16 hours. Peptides were extracted by carrying out three 20 minutes incubations in 40 μL of 60% acetonitrile dissolved in 0.5% HCOOH. The resulting peptide extracts were pooled, concentrated in a SpeedVac, and stored at −20 °C

### Protein quantification by SWATH-MS (Sequential Window Acquisition of all Theoretical Mass Spectra)

Since it is a recent technology SWATH-MS has been demonstrated to be a translational and a valuable tool in different fields being successfully applied by our group in a large variety of studies[4][5]

*Creation of the spectral library - Data-dependent acquisition (DDA)*

To construct the MS2 (MS/MS spectral libraries) spectral library, the peptide solutions from the discovery cohort were analysed by a shotgun **data-dependent acquisition (DDA)** approach by **micro-liquid chromatography-MS/MS.** To get a good representation of the peptides and proteins present in all samples, pooled vials of samples from each group (basal n=48, 6 weeks n=35, 12 weeks n=30 and progression disease, n=21) were prepared using equal mixtures of the original samples. Four μL (1 µg/μL) of the pool was separated using Reverse Phase Chromatography. Gradient was created using a micro liquid chromatography system (micro-LC) Ekspert nLC425 (Eksigent Technologies nanoLC 400, Sciex, CA, USA) coupled to high-speed Triple TOF 6600 mass spectrometer (Sciex, CA, USA) with a micro flow source. The chosen analytical column was a silica-based reversed phase column Chrom XP C18 150 × 0.30 mm, 3 mm particle size and 120 Å pore size (Eksigent, Sciex, CA, USA). The trap column was a YMC-TRIART C18 (YMC Technologies, Teknokroma) with a 3 mm particle size and 120 Å pore size, switched on-line with the analytical column. The loading pump delivered a solution of 0.1% formic acid in water at 10 µl/min. The micro-pump generated a flow-rate of 5 µl/min and was operated under gradient elution conditions, using 0.1% formic acid in water as mobile phase A, and 0.1% formic acid in acetonitrile as mobile phase B. Peptides was separated using a 90 minutes gradient ranging from 2% to 90% mobile phase B.

Data acquisition was performed in a TripleTOF 6600 System (Sciex, Foster City, CA) using a Data dependent workflow (DDA). Source and interface conditions was the following: ionspray voltage floating (ISVF) 5500 V, curtain gas (CUR) 25, collision energy (CE) 10 and ion source gas 1 (GS1) 25. Instrument was operated with Analyst TF 1.7.1 software (Sciex, USA). Switching criteria was set to ions greater than mass to charge ratio (m/z) 350 and smallerthan m/z 1400 with charge state of 2–5, mass tolerance 250 ppm and an abundance threshold of more than 200 counts (cps). Former target ions were excluded for 15 s. The instrument was automatically calibrated every 4 hours using as external calibrant tryptic peptides from PepCalMix.

*Data Analysis*

After MS/MS analysis (MS2 data), data files were processed using ProteinPilot^TM^ 5.0.1 software from Sciex which uses the algorithm Paragon^TM^ [6]for database search and Progroup^TM^ for data grouping. Data was searched using a Human specific Uniprot database (*UniProt release 2022_02 With 44413 human proteins*), specifying iodoacetamide as Cys alkylation as variable modification and methionine oxidation as fixed modification. False discovery rate was performed using a non linear fitting method displaying only those results that reported a1% Global false discovery rate or better [7][3] Only peptides with a confidence score above 99% (as obtained from Protein Pilot database search) were included in the spectral library from pooles (named poollibray). This library was created to obtain the fragment ion intensity and retention time dimensions essentially to make the SWATH method. In addition, a spectral online library called Human Pan-Human library, which contained 1,164,312 transitions identifying 139,449 proteotypic peptides and 12,046 proteins [8] were also employed in order to improve and expand the coverage of the identified NSCLC cancer plasma proteome in our library. The sum of the two libraries constitutes our final library, denominated NSClibrary. This combinatorial library will allow us to increase the identification rate of proteins compared to the traditional library and obtain better protein biomarkers that can be minoritary proteins that can be poured into plasma due to the disease process.

The mass spectrometry proteomics data have been deposited to the ProteomeXchange Consortium via the PRIDE[9] partner repository with the dataset identifier PXD042091

*Relative quantification by SWATH acquisition - DIA*

SWATH-MS acquisition was performed on a TripleTOF^®^ 6600 LC-MS/MS system (AB Sciex, CA, USA). Samples were analysed using **data-independent acquisition (IDA)** method (n=171 samples). Each sample (4 μL) was analysed using the liquid chromatography–mass spectrometry (LC-MS) equipment and LC gradient described above for building the spectral library but instead using the SWATH-MS acquisition method. The method consisted of repeating a cycle that is composed of the acquisition of 100 (MS/MS scans) MS2 scans (400 to 1500 m/z, high sensitivity mode, 50 ms acquisition time) of overlapping sequential precursor isolation windows of variable width (1 m/z overlap) covering the 400 to 1250 m/z mass range with a previous TOF MS MS1 scan (400 to 1500 m/z, 50 ms acquisition time) for each cycle. Total cycle time was 6.3 seconds. For the sample set, the width of the **100 variable windows** was optimised according to the ion density found in the DDA runs using a SWATH variable window calculator worksheet from Sciex. SWATH quantification was attempted for all proteins in the ion library that were identified by ProteinPilot with an FDR below 1%.

*Data analysis*

The targeted data extraction of the fragment ion chromatogram traces from the SWATH runs was performed by PeakView (version 2.2, Sciex) using the SWATH Acquisition MicroApp(version 2.0). This application processed the data using **NSClibrary**. PeakView computed an FDR and a score for each assigned peptide according to the chromatographic and spectra components; only peptides with an FDR below 1% were used for protein quantization; any shared and modified peptides were excluded from the processing. The retention times from the peptides that were selected for each protein was realigned in each run according to the iRT peptides corresponding to different identified proteins in each sample and eluted along the whole time axis. Five-minute windows and **30 ppm widths** were used to extract the ion chromatograms. Them extracted ion chromatograms were generated for each selected fragment ion; the peak areas for the protein were obtained by summing the peak areas from **10 peptides** (**MS1 scan) and 7 corresponding fragment ions/transitions (MS2 scan)** from each peptide.Them this integrated peak areas (processed. mrkvw files from PeakView) were directly exported to the MarkerView 1.3.1 software (AB Sciex, CA, USA) for relative quantitative analysis[4][5]. The export data will generate three files containing quantitative information about individual ions, the summed intensity of different ions for a particular peptide and the summed intensity of different peptides for a particular protein.

### Quality control and statistical analysis

Protein values derived from MarkerView 1.3.1 software (AB Sciex, CA, USA), were employed to perform the posterior statistical analysis. First, a quality control was performed. Thus, proteins with over 30% missing values in the sample set were filtered out. Second, missing values of the remaining proteins were imputed using the RandomForest R package[10][11]. Next, we normalised our values. Normalisation techniques are imperfect and may even introduce errors, especially if the data deviate from the assumptions of the chosen normalisation method. For this reason, we compared different quantile normalisation methods, in order to find the best option for our sample data set. We conducted additional analyses using diverse normalisation methods such as median normalisation, quantile normalisation, and LOESS normalisation (see Supplementary Figure 1), showing similar results. Quantile normalisation method, that is known for generating well-aligned distributions, ensuring that QN-normalized samples share identical distributions [12] was our final choice. With normalized data, student’s t test was applied to identify differentially expressed proteins (DEPs), with a *p*-value <0.05 and *p*-value <0.01 (no FDR correction). Finally, a final predictive model to separate responder group to non-responder group was developed following next steps: (1) **Model Building:** The normalised protein expression data was used for model building to discriminate between responder and non-responder groups. We employed Cox proportional hazards regression models, comparing them using the Akaike information criterion (AIC) technique[13] with a smaller AIC value indicating the better model. This approach identifies the model with the best fit based on the number of included variables and overall goodness-of-fit. (2) **Stepwise Backward Elimination:** To determine the optimal number of proteins for the panel, we performed a stepwise backward elimination procedure. Here, we iteratively removed the least informative protein (based on the highest p-value) from the model and recalculated the AIC. This process continued until the AIC reached a minimum, indicating the most informative subset of proteins for inclusion in the panel. (3) **Model Performance Evaluation:** The performance of the model with the 7-selected proteins was assessed using ROC curves. The AUC was calculated to quantify the model's ability to discriminate between responders and non-responders.

Continues data were compared using t test for independent samples and categorical variables were compared using Fisher's exact test. Swimmer plot was provided to visualise every patient's therapy response and the time of survival from the diagnoses. Kaplan-Meier method was used to plot the survival curves applying the log rank test. To identify the functions and relevant pathways of the DEPs, we performed gene ontology (GO) using Metascape[14]. The Kyoto Encyclopedia of Genes and Genomes (KEGG) pathway of each group was generated and visualize with proteomaps, using a web tool based on the t-test difference values without log2 transformation [15]. Receiver operating characteristic (ROC) curves were computed based on protein levels, representing the Area under the curve (AUC) values, and computing the confidence interval (CI) at 95% confidence levels. ROC curves were also constructed to evaluate the thresholds of baseline proteins levels for survival analyses. All statistical analyses were performed using GraphPad Prism version 8.0, IBM^®^ SPSS^®^ statistics version 25.0 and R version 4.1.1. The following R packages were used: survival [16] survminer, ggplot2[17] pROC [10] gtsummary [11] swimplot, RandomForest, MASS and stats.

## REFERENCES

1. de Jesus, J.R.; da Silva Fernandes, R.; de Souza Pessôa, G.; Raimundo, I.M.; Arruda, M.A.Z. Depleting high-abundant and enriching low-abundant proteins in human serum: An evaluation of sample preparation methods using magnetic nanoparticle, chemical depletion and immunoaffinity techniques. *Talanta* **2017**, *170*, 199–209, doi:10.1016/j.talanta.2017.03.091.

2. Fernández, C.; Santos, H.M.; Ruíz-Romero, C.; Blanco, F.J.; Capelo-Martínez, J.L. A comparison of depletion versus equalization for reducing high-abundance proteins in human serum. *Electrophoresis* **2011**, *32*, 2966–2974, doi:10.1002/elps.201100183.

3. Pereira-Veiga, T.; Bravo, S.; Gómez-Tato, A.; Yáñez-Gómez, C.; Abuín, C.; Varela, V.; Cueva, J.; Palacios, P.; Dávila-Ibáñez, A.B.; Piñeiro, R.; et al. Red Blood Cells Protein Profile Is Modified in Breast Cancer Patients. *Mol. Cell. Proteomics* **2022**, *21*, 100435, doi:10.1016/j.mcpro.2022.100435.

4. Ayuso-García, P.; Sánchez-Rueda, A.; Velasco-Avilés, S.; Tamayo-Caro, M.; Ferrer-Pinós, A.; Huarte-Sebastian, C.; Alvarez, V.; Riobello, C.; Jiménez-Vega, S.; Buendia, I.; et al. Neddylation orchestrates the complex transcriptional and posttranscriptional program that drives Schwann cell myelination. *Sci. Adv.* **2024**, *10*, eadm7600, doi:10.1126/sciadv.adm7600.

5. López-Valverde, L.; Vázquez-Mosquera, M.E.; Colón-Mejeras, C.; Bravo, S.B.; Barbosa-Gouveia, S.; Álvarez, J.V.; Sánchez-Martínez, R.; López-Mendoza, M.; López-Rodríguez, M.; Villacorta-Argüelles, E.; et al. Characterization of the plasma proteomic profile of Fabry disease: Potential sex- and clinical phenotype-specific biomarkers. *Transl. Res.* **2024**, *269*, 47–63, doi:10.1016/j.trsl.2024.02.006.

6. Shilov, I. V.; Seymourt, S.L.; Patel, A.A.; Loboda, A.; Tang, W.H.; Keating, S.P.; Hunter, C.L.; Nuwaysir, L.M.; Schaeffer, D.A. The paragon algorithm, a next generation search engine that uses sequence temperature values sequence temperature values and feature probabilities to identify peptides from tandem mass spectra. *Mol. Cell. Proteomics* **2007**, *6*, 1638–1655, doi:10.1074/mcp.T600050-MCP200.

7. Ortea, I.; Ruiz-Sánchez, I.; Cañete, R.; Caballero-Villarraso, J.; Cañete, M.D. Identification of candidate serum biomarkers of childhood-onset growth hormone deficiency using SWATH-MS and feature selection. *J. Proteomics* **2018**, *175*, 105–113, doi:10.1016/j.jprot.2018.01.003.

8. Rosenberger, G.; Koh, C.C.; Guo, T.; Röst, H.L.; Kouvonen, P.; Collins, B.C.; Heusel, M.; Liu, Y.; Caron, E.; Vichalkovski, A.; et al. A repository of assays to quantify 10,000 human proteins by SWATH-MS. *Sci. data* **2014**, *1*, 140031, doi:10.1038/sdata.2014.31.

9. Perez-Riverol, Y.; Bai, J.; Bandla, C.; García-Seisdedos, D.; Hewapathirana, S.; Kamatchinathan, S.; Kundu, D.J.; Prakash, A.; Frericks-Zipper, A.; Eisenacher, M.; et al. The PRIDE database resources in 2022: a hub for mass spectrometry-based proteomics evidences. *Nucleic Acids Res.* **2022**, *50*, D543–D552, doi:10.1093/nar/gkab1038.

10. Robin, X.; Turck, N.; Hainard, A.; Tiberti, N.; Lisacek, F.; Sanchez, J.-C.; Müller, M. pROC: an open-source package for R and S+ to analyze and compare ROC curves. *BMC Bioinformatics* **2011**, *12*, 77, doi:10.1186/1471-2105-12-77.

11. Sjoberg, Daniel, D.; Whiting, K.; Curry, M.; Lavery, Jessica, A.; Larmarange, J. Reproducible Summary Tables with the gtsummary Package. *R J.* **2021**, *13*, 570, doi:10.32614/RJ-2021-053.

12. Schmid, R.; Baum, P.; Ittrich, C.; Fundel-Clemens, K.; Huber, W.; Brors, B.; Eils, R.; Weith, A.; Mennerich, D.; Quast, K. Comparison of normalization methods for Illumina BeadChip HumanHT-12 v3. *BMC Genomics* **2010**, *11*, 349, doi:10.1186/1471-2164-11-349.

13. Akaike, H. A new look at the statistical model identification. *IEEE Trans. Automat. Contr.* **1974**, *19*, 716–723, doi:10.1109/TAC.1974.1100705.

14. Zhou, Y.; Zhou, B.; Pache, L.; Chang, M.; Khodabakhshi, A.H.; Tanaseichuk, O.; Benner, C.; Chanda, S.K. Metascape provides a biologist-oriented resource for the analysis of systems-level datasets. *Nat. Commun.* **2019**, *10*, 1523, doi:10.1038/s41467-019-09234-6.

15. Liebermeister, W.; Noor, E.; Flamholz, A.; Davidi, D.; Bernhardt, J.; Milo, R. Visual account of protein investment in cellular functions. *Proc. Natl. Acad. Sci.* **2014**, *111*, 8488–8493, doi:10.1073/pnas.1314810111.

16. Therneau, T.M.; Grambsch, P.M. *Modeling Survival Data: Extending the Cox Model*; Springer: New York, 2000; ISBN 0-387-98784-3.

17. Hadley, W. *Ggplot2: Elegrant graphics for data analysis*; Springer, 2016; ISBN 3319242776.

**SUPPLEMENTARY FIGURES**


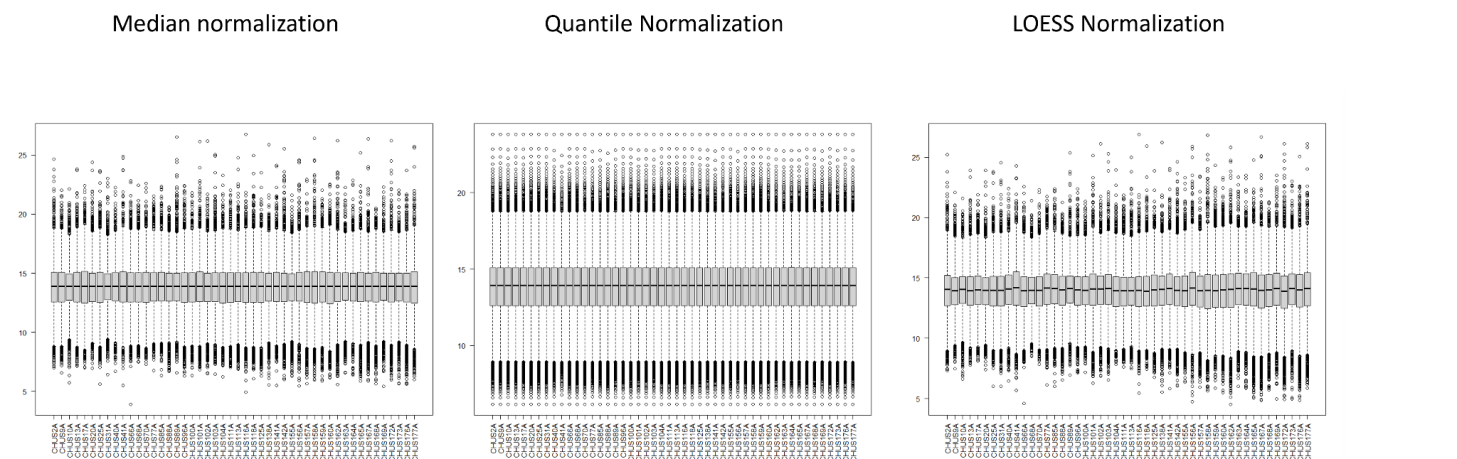


**Supplementary Figure 1**. Different normalisation methods such as median normalisation, quantile normalisation and LOESS normalisation were tested. These analyses consistently produced similar results. In our work, we chose to use quantile normalisation, which is particularly advantageous in proteomic analyses where protein abundance data often do not follow specific parametric distributions.


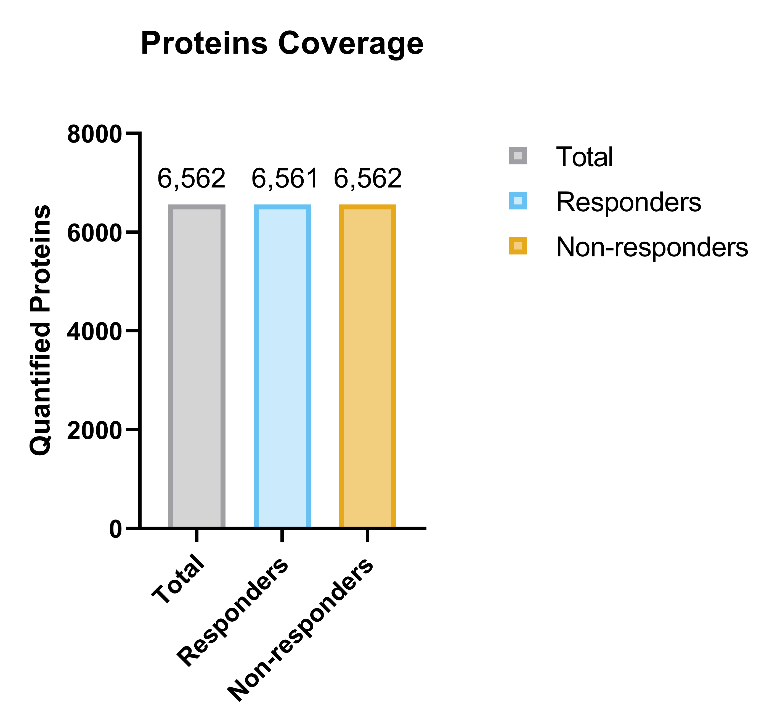


**Supplementary Figure 2**. Total number of proteins quantified in each group of patients by SWATH-MS technology. Abbreviations: R, responders; NR, non-responders.


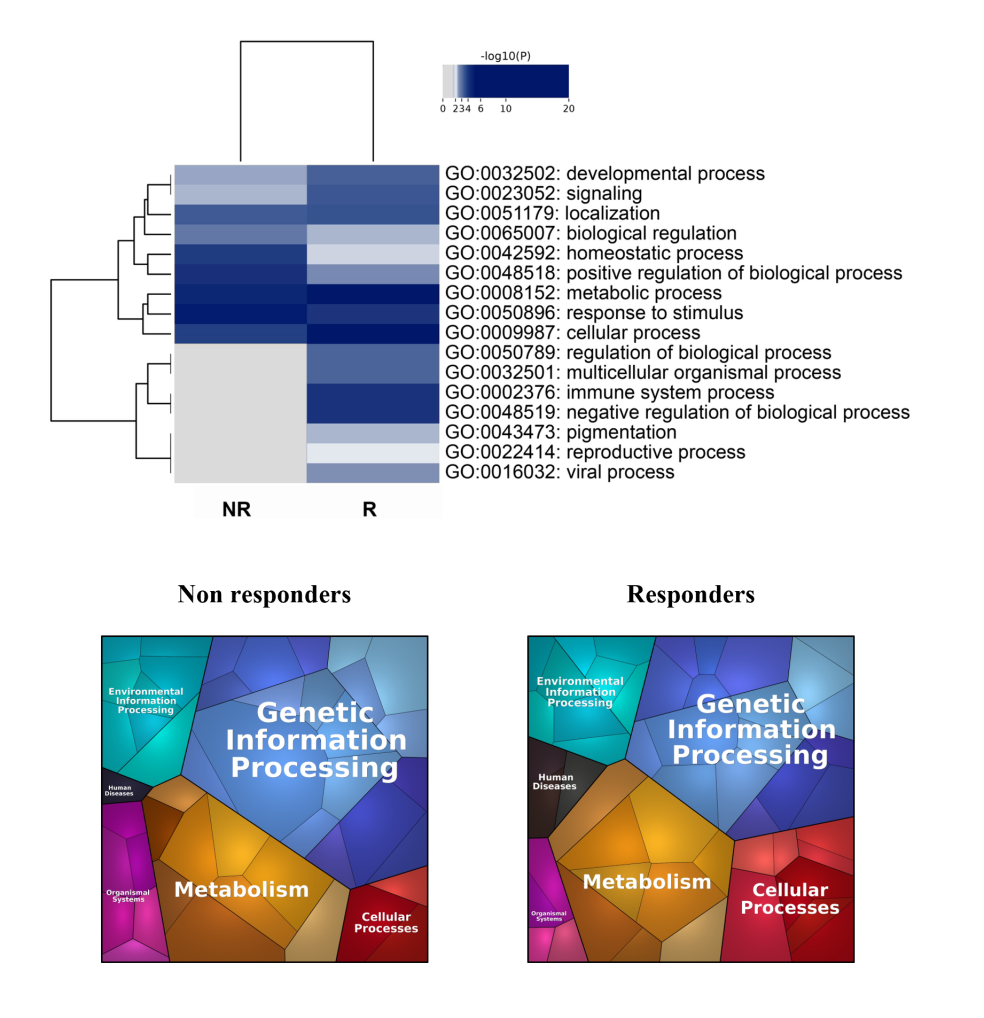


**Supplementary Figure 3.** Heatmap showing the top enrichment clusters, one row per group, using a colour scale to represent statistical significance. Gray colour indicates a lack of significance. Proteomaps that showed the functional differences between R (left) and NR (right) to immunotherapy. Each polygon corresponds to a single KEGG pathway, and its size correlates with the ratio between both groups.


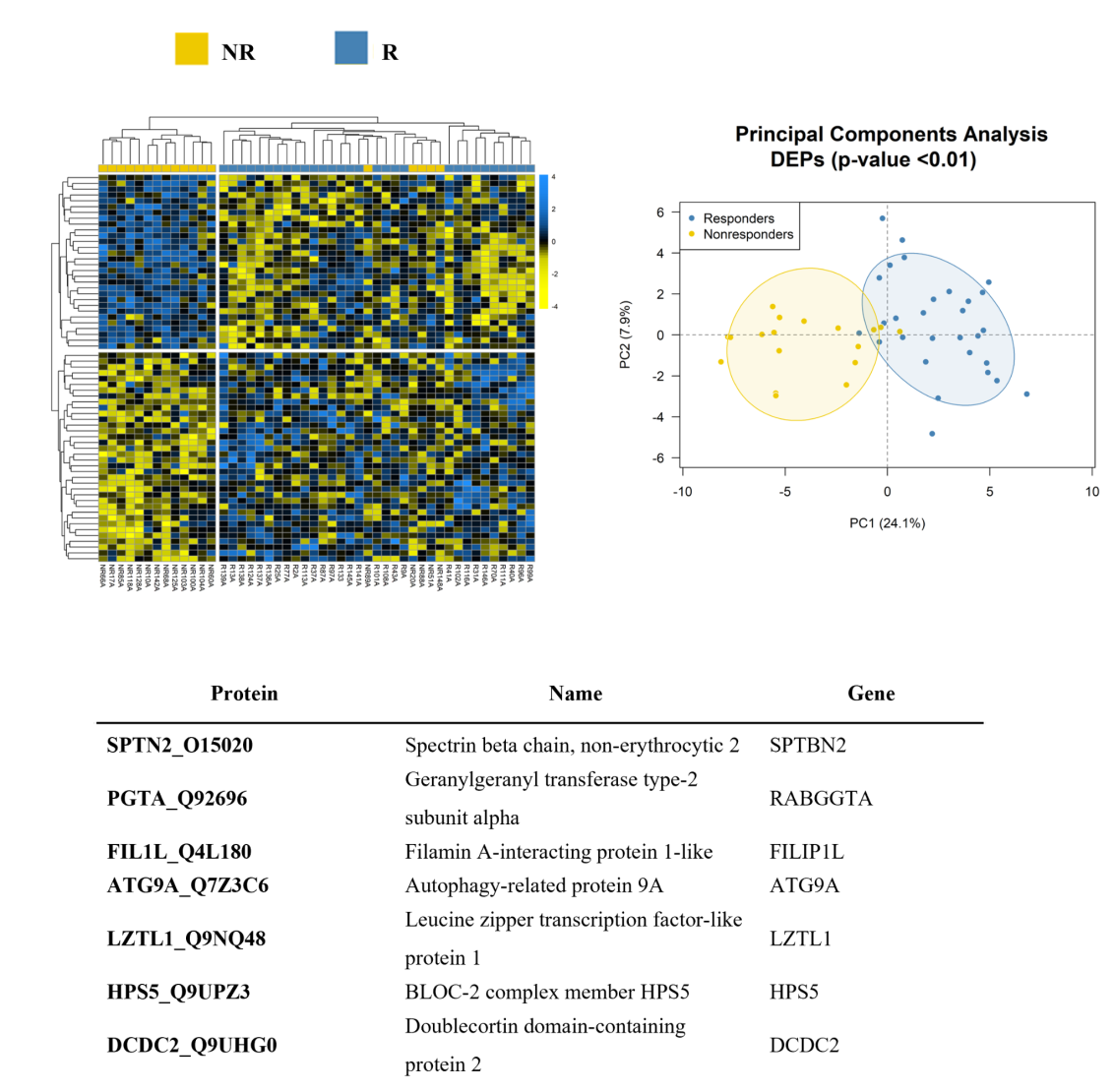


**Supplementary Figure 4**. (A) Heatmap of 66 differentially expressed proteins at baseline (*p-*value <0.01), that discriminate between R and NR patients to pembrolizumab therapy. (B) Principal Component Analysis (PCA) analysis showing the separation of samples from responders (blue) and non-responders (yellow) to pembrolizumab therapy according the 66 DEPs found by LC-MS/MS analysis. (C) Characteristics of the 7 proteins that allow us to predict immunotherapy response in advanced NSCLC patients.


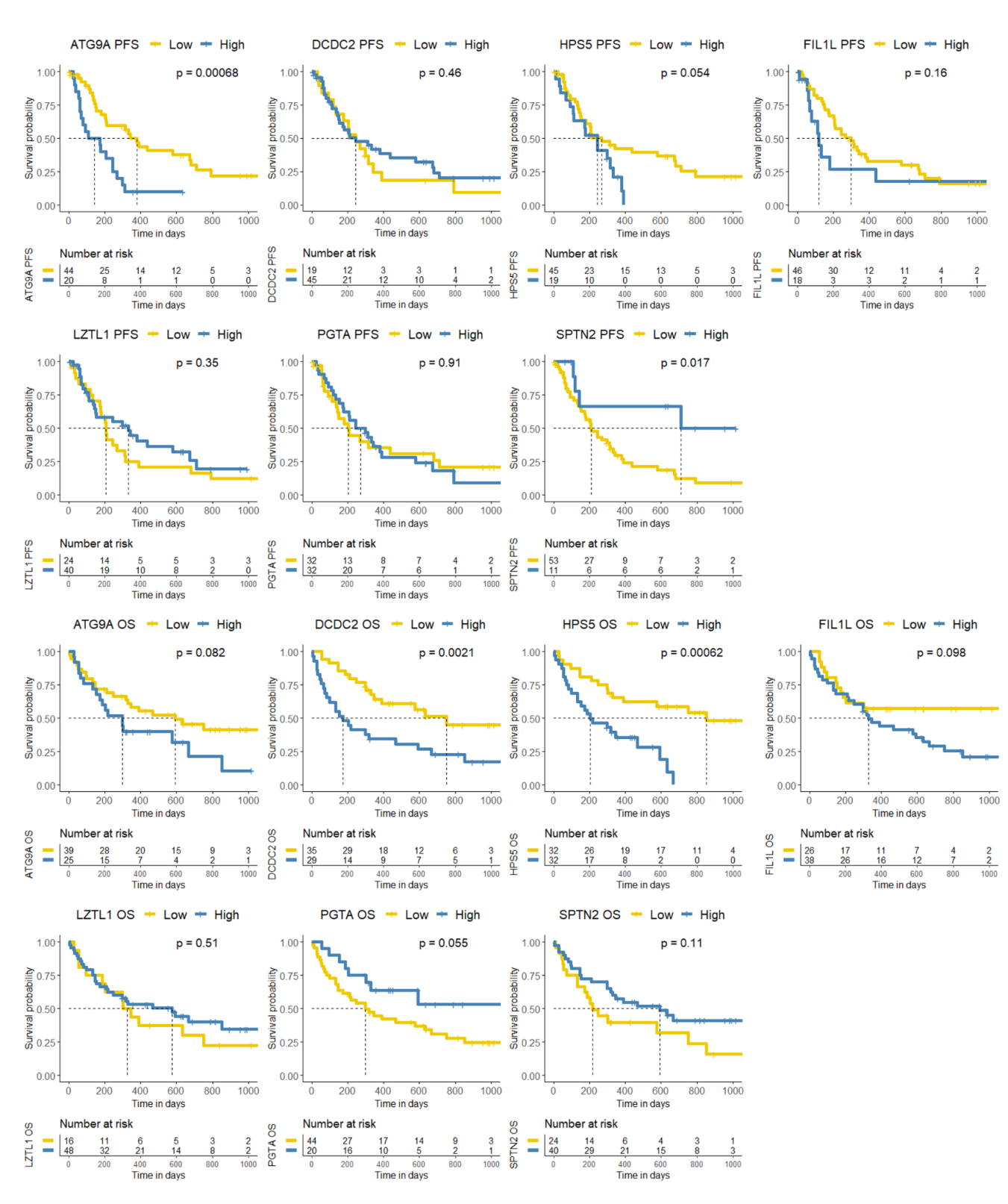


**Supplementary figure 5**. Kaplan-Meier survival analysis of ATG9A, DCDC2, FIL1L, PGTA, SPTN2 and HPS5 protein levels at baseline. Kaplan-Meier plots of PFS and OS did not show significant associations between protein levels before start therapy and prognosis in metastatic NSCLC under immunotherapy regimens.

**SUPPLEMENTARY TABLES**

**Supplementary Table 1**. Characteristics of the 7 proteins in the NSCLibrary that allow us to predict immunotherapy response in advanced NSCLC patients. The number of peptides assigned indicate the peptides that were matched with this protein The protein sequence coverage shows the percentage of the original protein sequences that are covered by identified peptide. The difference in these 7 proteins between responders and non-responders is shown by relevant quantitative data such as p-value and FC. Abbreviations: MSMS, Tandem mass spectrometry; FC, fold change.

| **Protein** | **Accession number** | **Nº of peptides assigned** | **MSMS** | **Protein sequence % coverage** | **Relevant quantitative data** | |
| --- | --- | --- | --- | --- | --- | --- |
|  |  |  |  |  | **p-value** | **FC** |
| SPTN2 | O15020 | 80 | 618 | 99% | 0.0012 | 1.115 |
| PGTA | Q92696 | 23 | 198 | 95% | 0.0010 | 1.0277 |
| FIL1L | Q4L180 | 10 | 61 | 95% | 0.0011 | -1.2926 |
| ATG9A | Q7Z3C6 | 19 | 144 | 100% | 0.0031 | -0.8367 |
| LZTL1 | Q9NQ48 | 11 | 73 | 92% | 0.0050 | -1.2941 |
| HPS5 | Q9UPZ3 | 7 | 48 | 100% | 0.0087 | -0.8511 |
| DCDC2 | Q9UHG0 | 3 | 19 | 100% | 0.0025 | -1.2630 |

**Supplementary Table 2.** Imputed Value Counts for Protein Models in the Discovery Cohort (n=48). The number of missing values of the seven proteins was taken into account to avoid any potential impact of imputation.

| **Protein** | **Accession number** | **% patients with imputed values (n)** |
| --- | --- | --- |
| SPTN2 | O15020 | 10.42 (n=5) |
| PGTA | Q92696 | 4.17 (n=2) |
| FIL1L | Q4L180 | 4.17 (n=2) |
| ATG9A | Q7Z3C6 | 0 (n=0) |
| LZTL1 | Q9NQ48 | 6.25 (n=3) |
| HPS5 | Q9UPZ3 | 0 (n=0) |
| DCDC2 | Q9UHG0 | 4.17 (n=2) |

**Supplementary Table 3.** AUC and confidence intervals for each protein model and their combination in the global cohort and stratified by cohort type. In the discovery cohort the AUC varies between 0.720 and 0.782, while in the validation cohort, the AUC varies between 0.483 and 0.683. In both cohorts, the AUC considering the 7 proteins, was 1. Abbreviations: AUC, area under the curve; CI, confidence intervals.

| **Protein** | **Global cohort, n=64**  **AUC (95% CI)** | **Discovery cohort, n=48**  **AUC (95% CI)** | **Validation cohort, n=16**  **AUC (95% CI)** |
| --- | --- | --- | --- |
| ATG9A | 0.705 (0.577-0.833) | 0.748 (0.604-0.893) | 0.600 (0.302-0.898) |
| DCDC2 | 0.657 (0.518-0.796) | 0.767 (0.627-0.907) | 0.483 (0.099-0.867) |
| FIL1L | 0.680 (0.543-0.817) | 0.754 (0.598-0.909) | 0.617 (0.262-0.971) |
| HPS5 | 0.686 (0.556-0.817) | 0.720 (0.572-0.869) | 0.600 (0.275-0.925) |
| LZTL1 | 0.648 (0.510-0.786) | 0.730 (0.578-0.881) | 0.583 (0.271-0.896) |
| PGTA | 0.729 (0.606-0.852) | 0.765 (0.631-0.899) | 0.683 (0.410-0.957) |
| SPTN2 | 0.636 (0.478-0.795) | 0.781 (0.639-0.924) | 0.750 (0.491-1) |
| 7-protein panel | 0.935 (0.876-0.995) | 1 (1.00 - 1.00) | 1 (1.00 - 1.00) |

**Supplementary Table 4.** AUC of individual proteins and combined 7-protein panel in the global cohort (n=64) and stratified by major histological subtypes: adenocarcinoma (n=52) vs. squamous cell carcinoma (n=10). There were 2 patients with other histologies, but these were not analysed separately due to the small size of the group. Abbreviations: AUC, area under the curve.

| **Proteins** | **All subtypes, n=64 AUC (95% CI)** | **ADC, n=52**  **AUC (95% CI)** | **SCC, n=10  AUC (95% CI)** |
| --- | --- | --- | --- |
| ATG9A | 0.705 (0.577-0.833) | 0.726 (0.586-0.867) | 0.708 (0.3493-1) |
| DCDC2 | 0.657 (0.518-0.796) | 0.695 (0.548-0.843) | 0.542 (0.136-0.947) |
| FIL1L | 0.680 (0.543-0.817) | 0.728 (0.579-0.877) | 0.583 (0.179-0.988) |
| HPS5 | 0.686 (0.556-0.817) | 0.686 (0.540-0.832) | 0.75 (0.400-1) |
| LZTL1 | 0.648 (0.510-0.786) | 0.719 (0.576-0.862) | 0.625 (0.211-1) |
| PGTA | 0.729 (0.606-0.852) | 0.727 (0.590-0.863) | 0.75 (0.390-1) |
| SPTN2 | 0.636 (0.478-0.795) | 0.672 (0.498-0.846) | 0.375 (0-0.836) |
| 7-protein panel | 0.935 (0.876-0.995) | 0.953 (0.903-1) | 1 (1.00-1.00) |

**Supplementary Table 5.** Receiver operating characteristic curves analysis to determine the cut-off of protein levels to discriminate progression or death. Proteins levels above the threshold were considered “High”. Protein levels below the threshold were considered “Low”.

|  | **PFS** | **OS** |
| --- | --- | --- |
| **Proteins** | **Threshold** | **Threshold** |
| ATG9A | 1.253715 | 1.215786 |
| DCDC2 | 1.325231 | 1.471992 |
| FIL1L | 1.360772 | 1.234946 |
| HPS5 | 1.384833 | 1.236492 |
| LZTL1 | 1.347378 | 1.244072 |
| PGTA | 1.230732 | 1.306816 |
| SPTN2 | 1.432393 | 1.305443 |

Abbreviations: PFS, progression free survival; OS, overall survival.
